# Exploring diseases burden in HIV population: Results from the CHAO (Comorbidities in HIV/AIDS outpatients) cross-sectional study in Kenya

**DOI:** 10.1016/j.gloepi.2024.100174

**Published:** 2024-10-22

**Authors:** Fausto Ciccacci, Benjamin Welu, Harrison Ndoi, Claudia Mosconi, Carolina De Santo, Mariachiara Carestia, Anna Maria Doro Altan, Joseph Murungi, Koome Muthuri, Mariagrazia Cicala, Giovanni Guidotti, Stefano Orlando

**Affiliations:** a Department of Biomedicine and Prevention, University of Rome Tor Vergata, Rome, Italy; b DREAM Program, Community of Sant'Egidio, Kenya; c DREAM Program, Community of Sant'Egidio, Rome, Italy; d County AIDS&STI Coordination, Meru, Kenya; e County Direction of Health Services, Meru, Kenya; f ASL Rome 1, Rome, Italy

**Keywords:** HIV, Comorbidities, NCDs, Epidemiological transition, Double burden of diseases

## Abstract

**Background:**

Africa is the epicenter of the HIV epidemic, with over two-thirds of the global population living with HIV. It is also facing a dramatic increase in non-communicable diseases (NCDs) amidst its aging population. This necessitates a healthcare approach that addresses both infectious diseases and NCDs in HIV-positive individuals. In Kenya, with 1.4 million HIV-positive people, efforts include widespread ART access and integrating HIV services into the health system. Challenges remain in healthcare infrastructure, particularly in rural areas. The Comorbidities in HIV/AIDS Outpatients (CHAO) Project, funded by the Italian Cooperation Agency, aims to improve understanding and management of comorbidities in HIV patients, highlighting the need for cost-effective healthcare strategies to address this dual burden.

**Methods:**

The CHAO (Comorbidities in HIV/AIDS Outpatients) project conducted a cross-sectional epidemiological study across 25 clinics in Meru County, Kenya. The study included comprehensive surveys and screenings for various comorbidities among HIV-positive patients receiving treatment, utilizing both clinical evaluations and laboratory tests to assess the prevalence of infectious diseases and NCDs.

**Results:**

A total of 1051 HIV-positive individuals were included in the study: 75 % females, 25 % males, median age 47 years, the majority (96 %) on long-term ART, mostly Dolutegravir-based (95 %). 55.4 % had at least one comorbidity, with NCDs such as dyslipidemia (21.22 %) and hypertension (20.17 %) being the most prevalent. The study also noted significant occurrences of communicable diseases, including syphilis (5.23 %), hepatitis B (2.19 %), and hepatitis C (0.29 %). The prevalence of comorbidities varied with age, highlighting the impact of aging on disease burden.

**Discussion:**

The high prevalence of comorbidities among HIV-positive patients in Meru County underscores the need for integrated healthcare strategies that address both infectious diseases and NCDs. The findings advocate for systematic screening and management of comorbidities within HIV care programs, emphasizing the need for holistic health approaches to improve outcomes for this population.

## Introduction

Africa remains at the epicenter of the global human immunodeficiency virus (HIV) epidemic, representing the region with the largest number of individuals living with HIV, over two-thirds of the global HIV-infected population [1]. In recent years, African countries have been undergoing significant changes, marked by the aging of their populations and an increase in non-communicable diseases (NCDs) [2,3]. These evolving demographic and health trends are leading to a specific double burden of disease among HIV patients on the continent [4,5]. Beyond the traditional risk of infectious diseases such as tuberculosis (TB) and syphilis, there is a noticeable rise in the prevalence of NCDs across many African countries [[6], [7], [8]]. This emerging dual burden of disease necessitates a healthcare approach in Africa that adeptly addresses both the long-standing challenge of infectious diseases and the growing prevalence of NCDs in HIV-positive populations [7,9,10]. This evolving scenario highlights the need for enhanced understanding and research on NCDs in African contexts, critical for developing effective healthcare strategies that address both infectious diseases and the growing impact of NCDs in HIV-infected individuals. This shift necessitates a reevaluation of healthcare strategies to address the dual burden of infectious diseases and chronic NCDs in this unique patient demographic [11].

In the context of Africa's diverse health challenges, the situation in Kenya provides a specific example, offering detailed insights into the local epidemiology. Data from the Joint United Nations Programme on HIV/AIDS (UNAIDS) indicates that around 1.4 million people in Kenya are living with HIV in 2023, with a national HIV prevalence rate of approximately 3.7 %, representing a significant public health concern [12]. Kenya's response to HIV is articulated, including widespread public awareness campaigns, increased access to antiretroviral therapy (ART), and integration of HIV services into the general health system. Despite these efforts, challenges persist, particularly in healthcare infrastructure and access, especially in rural and underserved areas. As of 2022, about 95 % of adults and 85 % of children living with HIV in Kenya were accessing ART [12]. With a large population of HIV patients, many of whom are on therapy and living longer, Kenya faces emerging scenarios that intertwine infectious diseases (such as TB, malaria, viral hepatitis, syphilis, etc.) with chronic NCDs.

Key national documents, including the Kenya AIDS Strategic Framework (KASF) and the Kenya HIV & AIDS Research Agenda, highlight significant gaps in understanding and managing these comorbidities. KASF is a national strategy aimed at reducing HIV infections and improving the lives of those affected by HIV/AIDS [13,14]; both KASF 1 (2013) and KASF 2 (2019) underscore the need for improved screening, prophylaxis, and management of co-infections and comorbidities, recommending comprehensive service packages encompassing various health checks and screenings. Additionally, the Kenya HIV & AIDS Research Agenda stresses the urgency of enhancing epidemiological data for effective healthcare planning, as current data on comorbidities in HIV patients is notably insufficient [15].

The Comorbidities in HIV/AIDS Outpatients (CHAO) Project in Meru County, Kenya, funded by the Italian Cooperation Agency (AICS) through the “Global Fund” initiative, aims to enhance understanding of comorbidities and key risk factors in HIV patients. Focused on identifying primary comorbidities in HIV-positive individuals, the project emphasizes the importance of recognizing additional health issues in patients under regular treatment and healthcare supervision. These often-overlooked comorbidities, if untreated, can lead to serious complications. The CHAO Project seeks to highlight the benefits of cost-effective diagnostic and therapeutic interventions in improving life quality for HIV patients in treatment.

## Methods

### Study design

The CHAO project is an epidemiological cross-sectional study conducted in Meru County, Kenya, aimed to ascertain the prevalence of various comorbidities among HIV-positive patients. Meru County was selected due to the implementing partners' prior experience in the area [16,17].

The study involved a comprehensive survey across 25 clinics offering HIV treatment. All the primary clinics offering HIV treatment in the county were included to ensure a comprehensive geographical coverage. The cross-sectional design facilitated the assessment of comorbidity prevalence among the patient population at a specific point in time.

One or more screening session were held in each clinic in the period January–June 2023.

The study strictly followed ethical guidelines to ensure confidentiality, informed consent, and voluntary participation. Ethical approval was secured from the African Medical and Research Foundation (AMREF) Ethical And Scientific Review Committee (ESRC) (P1201.2022) and licensed by the National Commission for Science, Technology and Innovation (NACOSTI) (P/22/19204), with endorsement from the Meru County Department of Health. Each patient was provided with detailed information about the study, along with reading materials in both English and Swahili, to ensure comprehensive understanding. Subsequently, patients who agreed to participate were requested to sign a consent form, affirming their willingness to be involved in the research.

### Participants

The study targeted all HIV-positive patients over 18 years present at the clinics during screening sessions. This inclusive approach aimed to capture a diverse and representative sample of the patient population with no specific choice by the study personnel. Inclusion criteria were: HIV positive patients in care in the clinic, age ≥ 18 years. Exclusion criteria was age < 18 years.

### Procedures

Data collection included gathering general information (age, sex), conducting clinical evaluations (physical examinations, vital signs, TB symptom screening), and performing laboratory tests (for hepatitis, syphilis, diabetes, dyslipidemia), moreover, information regarding lifestyle factors (tobacco and alcohol consumption), medical history (duration since HIV diagnosis, ART regimen) were collected. Tobacco consumption was assessed through self-reporting, with participants indicating whether they were current smokers, recent smokers (within the past year), or had never smoked; no quantification of pack years was performed. Alcohol consumption was similarly assessed through a binary self-report question, where participants were asked if they consumed alcohol (yes/no), without further quantification or categorization of drinking patterns.

Alongside general information and clinical evaluations, patients were measured on-site for anthropometric measurements and risk factors, including height, weight, and blood pressure. When elevated blood pressure was measured, the measurement was repeated three times, and the lowest value was recorded. Laboratory samples were collected at these sessions. These samples were then analyzed at the DREAM laboratories in Consolata Hospital (Nkubu) and in the Aina Dispensary.

Samples collected included blood samples for Full blood count, Liver transaminases, Lipid profile (high density lipoproteins (HDL), low density lipoproteins (LDL) and Triglycerides) and Creatinine levels. Rapid tests were done on site for fasting blood glucose, Hepatitis B and C, venereal disease research laboratory (VDRL) for syphilis. Patients with symptoms of TB were referred to referral centers for diagnosis and treatment.

### Outcomes

The primary outcome was the presence of different comorbidities within the screened population.

The cutoff and diagnostic criteria used were as follows: nutritional status was evaluated using the Body Mass Index (BMI) and categorized as underweight (BMI < 18.5 kg/m^2^), normal weight (18.5 ≤ BMI < 24.9 kg/m^2^), overweight (25 ≤ BMI < 29.9 kg/m^2^), obesity (BMI ≥ 30 kg/m^2^) [18]; blood pressure was categorized as optimal (systolic blood pressure (SBP) < 120 mmHg, diastolic blood pressure (DBP) < 80 mmHg), normal (120 ≤ SBP ≤ 129 mmHg, 80 ≤ DBP ≤ 84 mmHg), high normal (130 ≤ SBP ≤ 139 mmHg, 85 ≤ DBP ≤ 89 mmHg), grade 1 hypertension (140 ≤ SBP ≤ 159 mmHg, 90 ≤ DBP ≤ 99 mmHg), grade 2 hypertension (160 ≤ SBP ≤ 179 mmHg, 100 ≤ DBP ≤ 109 mmHg), grade 3 hypertension (SBP ≥ 180 mmHg, DBP ≥ 110 mmHg), isolated systolic hypertension (SBP ≥ 140 mmHg, DBP < 90 mmHg) [19]; random blood glucose (RBG) was considered normal <11.1 mmol/L, patients with higher RBG were considered diabetic [20]; haemoglobin (Hbg) was categorized as normal (Hbg >12 g/dL in women and > 13 g/dL in man), mild anemia (10 ≤ Hbg < 12 g/dL in women and 10 ≤ Hbg < 13 g/dL in men), moderate anemia (8 ≤ Hbg < 10 g/dL both in women and men), and severe anemia (Hbg < 8 g/dL) [21]; hypertransaminasemia included moderate elevation (ALT or AST levels 5–15 times the upper limit of 38 U/L in women and 43 U/L in men for ALT, and 32 U/L for women and 40 U/L for men for AST) and severe elevation (ALT or AST levels are more than 15 times the upper limit) [22]; patients were categorized as dyslipidemic if cholesterol was elevated (> 240 mg/dL) and/or triglycerides were elevated (>200 mg/dL) or borderline (150-200 mg/dL) [23]; creatininemia was considered normal if <1.2 mg/dL; eGFR (estimated Glomerular Filtration Rate) was calculated using the 4-variable equation from the Modification of Diet in Renal Disease (MDRD) Study and was categorized as normal (eGFR >90 mL/min), mild impairment (eGFR 90–60 mLl/min), moderate impairment (eGFR 60–30 mL/min), and severe impairment (eGFR <30 mL/min) [24] .

For HBV, Hepatitis B surface antigen rapid test results were reported as positive or negative. Similarly, results of HCV antigen test were also reported as positive or negative. Rapid test results for syphilis (VDRL) were similarly reported as positive and negative.

Following the classification used by the Global Burden of Disease study [25], the diseases investigated in this study were grouped into two main categories: a) NCDs and b) communicable, maternal, perinatal and nutritional (including only undernutrition) conditions. The NCDs included hypertension, diabetes, dyslipidemias, obesity and overweight conditions. As for communicable diseases, we considered syphilis, Hepatitis C (HCV), Hepatitis B (HBV), suspected TB, and undernutrition.

### Statistical analysis

The minimum sufficient sample size was determined to investigate the prevalence of comorbidities among HIV-positive patients. We applied the formula n = (Z^2^ * P(1-P))/E^2^ , with Z = 1.96 for 95 % confidence, and a 5 % margin of error (E = 0.05); given the wide range of reported NCD prevalence in diverse populations, we selected a median estimate of 11 % as a realistic average figure for our study's baseline prevalence rate (*P* = 0.11); with this calculation we determined a base sample size of 151 subjects. For a detailed analysis across two gender groups and three age groups, we multiplied the initial estimate by six, reflecting the six demographic strata, resulting in 904 subjects. To account for possible discrepancies in data collection and enhance the study's validity, we ultimately expanded our sample to 1000 individuals.

We conducted descriptive statistical analysis to characterize the sample population, detailing the distribution of key demographic and clinical variables. Additionally, we performed a univariate analysis and a multivariable logistic regression to assess the various risk factors under investigation. The multivariable model included the following variables: gender, age, time since HIV diagnosis, tobacco use, and alcohol use. Overweight and obesity were specifically evaluated as potential risk factors for NCDs, while malnutrition was examined as an outcome variable. The data analysis was performed using R Studio (version 4.3.1), focusing on descriptive statistics (including frequencies, percentages, medians, and interquartile ranges).

## Results

The cohort comprised 1051 HIV-positive individuals, with a female predominance of 75 % (*n* = 791) and males constituting 25 % (*n* = 260). Table 1 show the characteristics of the sample and blood test results. The median age was 47 years (range: 38–54 years). Patients reported tobacco use in 7.3 % (*n* = 77) and alcohol consumption in 15 % (*n* = 151).

**Table 1:** General and anthropometric characteristics of the HIV patients and blood test results, for the total sample and for females and males patients.

	Total sample *N* = 1051^1^	Females *N* = 791^1^	Males *N* = 260^1^
Age (years)	47 (38, 54)	46 (38, 53)	49 (42, 57)
Unknown	2	1	1
Tobacco consumers	77 (7.3 %)	20 (2.5 %)	57 (22 %)
Unknown	3	3	0
Alcohol consumers	151 (15 %)	59 (8 %)	92 (38 %)
Unknown	67	49	18
Time from HIV diagnosis			
> 12 months	1000 (96 %)	749 (96 %)	251 (97 %)
6–12 months	18 (1.7 %)	15 (1.9 %)	3 (1.2 %)
< 6 months	25 (2.4 %)	20 (2.6 %)	5 (1.9 %)
Unknown	8	7	1
Antiretroviral Treatment regimen			
With dolutegravir	998 (95 %)	753 (95 %)	245 (94 %)
Without dolutegravir	49 (4.7 %)	35 (4.4 %)	14 (5.4 %)
Unknown	4 (0.4 %)	3 (0.4 %)	1 (0.4 %)
Antiretroviral Treatment regimen line			
1st Line	1003 (96 %)	755 (96 %)	248 (96 %)
2nd Line	45 (4.3 %)	34 (4.3 %)	11 (4.2 %)
Unknown	3	2	1
Nutritional status			
Underweight	167 (16 %)	100 (13 %)	67 (26 %)
Normal weight	564 (54 %)	410 (52 %)	154 (59 %)
Overweight	223 (21 %)	197 (25 %)	26 (10 %)
Obese	97 (9.2 %)	84 (11 %)	13 (5.0 %)
Known hypertensive			
Newly diagnosed	100 (9.6 %)	72 (9.2 %)	28 (11 %)
No	834 (80 %)	628 (80 %)	206 (80 %)
Yes/Not on treatment	5 (0.5 %)	4 (0.5 %)	1 (0.4 %)
Yes/On treatment	105 (10 %)	81 (10 %)	24 (9.3 %)
Unknown	7	6	1
Known diabetic			
No	1018 (98 %)	761 (98 %)	257 (99 %)
Yes /on treatment	15 (1.4 %)	14 (1.8 %)	1 (0.4 %)
Yes/not on treatment	2 (0.2 %)	1 (0.1 %)	1 (0.4 %)
Unknown	16	15	1
Blood pressure			
Optimal	355 (34 %)	269 (34 %)	86 (33 %)
Normal	348 (33 %)	262 (33 %)	86 (33 %)
High normal	182 (17 %)	139 (18 %)	43 (17 %)
Grade 1 hypertension	120 (11 %)	85 (11 %)	35 (14 %)
Grade 2 hypertension	27 (2.6 %)	22 (2.8 %)	5 (1.9 %)
Grade 3 hypertension	13 (1.2 %)	11 (1.4 %)	2 (0.8 %)
Unknown	6	3	3
GPT class			
Normal	859 (82 %)	645 (82 %)	214 (83 %)
Mild	186 (18 %)	142 (18 %)	44 (17 %)
Moderate	1 (<0.1 %)	1 (0.1 %)	0 (0 %)
Elevated	0 (0 %)	0 (0 %)	0 (0 %)
Unknown	5	3	2
GOT class			
Normal	571 (55 %)	396 (50 %)	175 (68 %)
Mild	473 (45 %)	390 (49 %)	83 (32 %)
Moderate	2 (0.2 %)	2 (0.3 %)	0 (0 %)
Elevated	0 (0 %)	0 (0 %)	0 (0 %)
Unknown	5	3	2
Cholesterol			
Normal	932 (89 %)	699 (88 %)	233 (90 %)
Borderline	85 (8.1 %)	66 (8.3 %)	19 (7.3 %)
Elevated	34 (3.2 %)	26 (3.3 %)	8 (3.1 %)
Triglycerides			
Normal	838 (80 %)	636 (80 %)	202 (78 %)
Borderline	110 (10 %)	78 (9.9 %)	32 (12 %)
Elevated	103 (9.8 %)	77 (9.7 %)	26 (10 %)
Creatinine			
Normal	751 (71 %)	582 (74 %)	169 (65 %)
Elevated	300 (29 %)	209 (26 %)	91 (35 %)
eGFR Classification (Kidney disease)			
Normal	287 (28 %)	198 (25 %)	89 (35 %)
Mild	407 (39 %)	282 (36 %)	125 (49 %)
Moderate	338 (32 %)	296 (38 %)	42 (16 %)
Severe	10 (1.0 %)	9 (1.1 %)	1 (0.4 %)
Unknown	9	6	3
Random blood sugar			
Normal	1024 (98 %)	770 (98 %)	254 (99 %)
Elevated	18 (1.7 %)	15 (1.9 %)	3 (1.2 %)
Unknown	9	6	3
HBSag			
Negative	1023 (98 %)	766 (97 %)	257 (99 %)
Positive	23 (2.2 %)	21 (2.7 %)	2 (0.8 %)
Unknown	5	4	1
HCV ag			
Negative	1042 (100 %)	784 (100 %)	258 (99 %)
Positive	3 (0.3 %)	1 (0.1 %)	2 (0.8 %)
Unknown	6	6	0
Syphilis			
Negative	992 (95 %)	746 (95 %)	246 (95 %)
Positive	55 (5.3 %)	41 (5.2 %)	14 (5.4 %)
Unknown	4	4	0
Haemoglobin			
Normal	707 (69 %)	524 (68 %)	183 (72 %)
Mild anemia	244 (24 %)	180 (23 %)	64 (25 %)
Moderate anemia	47 (4.6 %)	43 (5.6 %)	4 (1.6 %)
Severe anemia	26 (2.5 %)	23 (3.0 %)	3 (1.2 %)
Unknown	27	21	6

Most participants (96 %, *n* = 1000) were on long-term ART, predominantly Dolutegravir based protocol (DTG) (95 %, *n* = 998), with 96 % (*n* = 1003) on a first-line regimen.

**Fig. 1:**
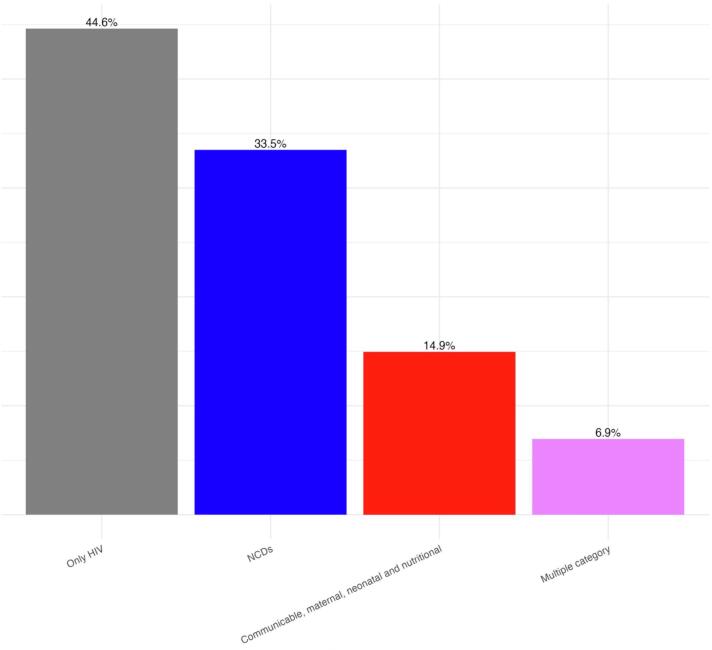
Proportion of patients affected by only HIV, HIV and non-communicable diseases, (NCDs) HIV and communicable, maternal, neonatal or nutritional conditions, and HIV and both NCDs and communicable, maternal, neonatal or nutritional conditions.

Table 1 also presents the different distributions of the studied variables by sex, highlighting that tobacco use was significantly higher among males (22 %, *n* = 57) compared to females (2.5 %, *n* = 20), and alcohol consumption was also more prevalent among males (38 %, *n* = 92) than females (8 %, *n* = 59). Additionally, Table 1 also reports the detailed results of the blood tests performed and possible missing results. Most patients (*n* = 582, 55.4 %) were affected by one or more comorbidity: 36.9 % had one, and 18.5 % had more than one comorbidity. As shown in Fig. 1, 352 patients (33.5 %) were affected by one or more NCD, 157 patients (14.9 %) by one or more communicable, and 73 patients (6.9 %) by both NCDs and communicable diseases. As the whole, NCDs affected 38.5 % of the sample, while communicable diseases 14.9 %.

**Fig. 2:**
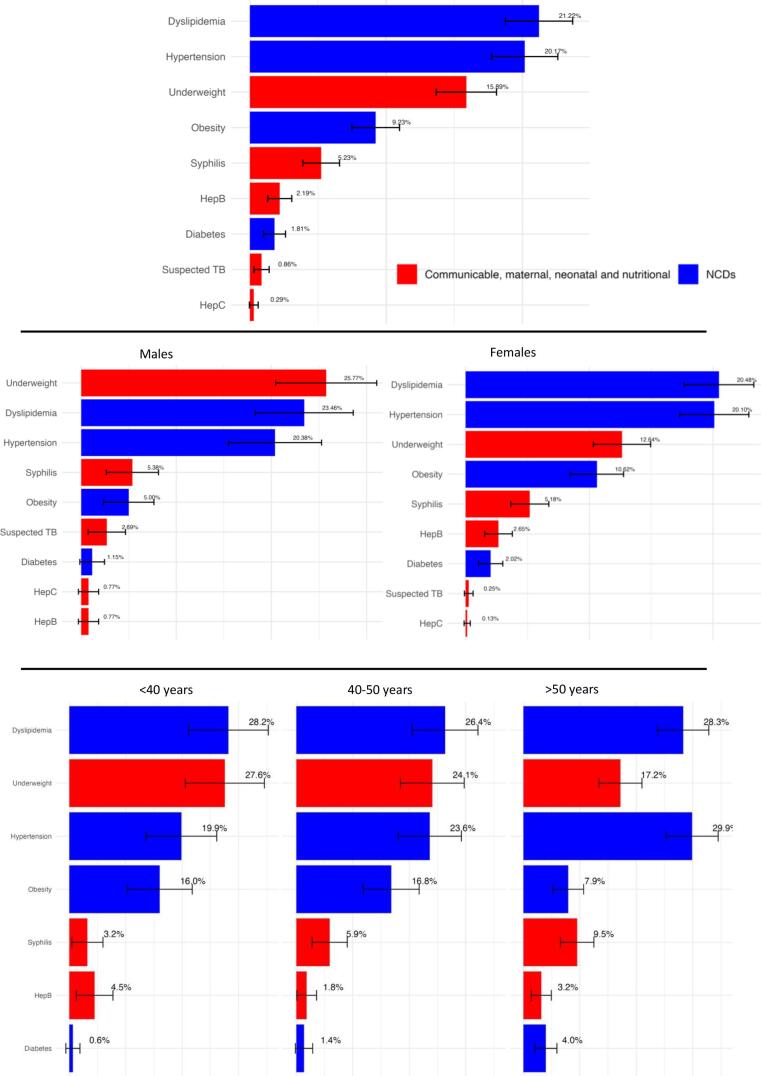
Comorbidity prevalence in the whole sample, for sex and for age groups.

Analysis of comorbidity prevalence (Fig. 2) revealed dyslipidemia and hypertension as the most prevalent non-communicable diseases (NCDs), with occurrences in 223 (21.22 %) and 210 (20.1 %) patients respectively. Among the 210 patients with hypertension, 100 were newly diagnosed during the screening sessions.

Underweight affected 167 patients (15.89 %). The prevalence of communicable diseases such as syphilis (*n* = 55, 5.23 %), hepatitis B (*n* = 23 2.19 %), and hepatitis C (*n* = 3, 0.29 %) was lower. Overweight or obesity were observed in 97 (9.23 %) patients, indicating a less prominent but still relevant concern for potential comorbidity in this demographic.

Fig. 2 also show some differences in the distribution of comorbidities between the two sexes. Women exhibited a higher prevalence of obesity/overweight (10.52 % vs 5 %), diabetes (2.02 % vs 1.15 %) and hepatitis B (2.65 % vs 0.77 %) compared to men, while higher prevalence of underweight (25.77 % vs 12.64 %), and suspected TB (2.69 % vs 0.25 %) were observed among males.

Comorbidity distribution varied with age as shown in Fig. 2, where age is categorized into three groups (<40, 40–50, and > 50) to reflect the distribution within our sample. Hypertension prevalence escalated from 19.9 % in individuals under 40 to 29.9 % in those over 50. The overweight and obesity proportion halved from 16.0 % in younger adults to 7.9 % in seniors. Contrastingly, underweight prevalence was higher in the younger cohort at 27.6 %, decreasing to 17.2 % over 50. Diabetes prevalence rose from 0.6 % in the youngest age group to 4 % in those over 50, underscoring the increasing burden of metabolic disorders with advancing age.

The multivariable analysis assessed independent risk factors for various conditions as reported in Table 2.

**Table 2:** Multivariable logistic regression results for the communicable and non-communicable diseases studied and selected characteristics (nutritional status (overweight) was considered as a risk factor only for non-communicable diseases and an outcome for the communicable diseases (undernutrition)).

		Male Sex	Age (years)			Time from HIV diagnosis			Tobacco consumers	Alcohol consumers	Overweight/obesity
			<40 years	40–50 years	>50 years	> 12 months	6–12 months	< 6 months			
1 or more comorbidities	OR 95 % CI	**1.48** **[1.05–2.09]**	–	1.39 [0.99–1.94]	**2.69** **[1.92–3.78]**	–	0.73 [0.23–2.18]	1.47 [0.61–3.68]	0.78 [0.42–1.45]	**1.69** **[1.08–2.67]**	–
Hypertension	OR 95 % CI	1.21 [0.79–1.84]	–	1.41 [0.87–2.33]	**3.50** **[2.24–5.59]**	–	1.46 [0.31–5.10]	0.61 [0.10–2.20]	0.75 [0.32–1.63]	0.97 [0.55–1.66]	**2.49** **[1.76–3.54]**
Diabetes	OR 95 % CI	0.76 [0.17–2.47]	–	2.37 [0.30–48.5]	**13.1** **[2.51–241]**	–	8.68 [0.42–64.5]	0.00 [0-inf]	0.00 [0-inf]	0.42 [0.02–2.42]	**3.66** **[1.42–10.2]**
Dyslipidemia	OR 95 % CI	1.19 [0.80–1.78]	–	1.01 [0.65–1.59]	**2.06** **[1.38–3.12]**	–	1.21 [0.27–4.02]	1.01 [0.29–2.82]	0.72 [0.34–1.47]	1.30 [0.78–2.13]	1.34 [0.95–1.90]
Obesity	OR 95 % CI	0.61 [0.30–1.14]	–	1.06 [0.60–1.87]	0.85 [0.48–1.52]	–	0.00	0.00	0.20 [0.01–1.08]	0.80 [0.31–1.75]	–
Suspected TB	OR 95 % CI	1.90 [0.30–16.4]	–	0.49 [0.06–3.17]	0.43 [0.05–2.83]	–	0.00	0.00	2.68 [0.47–19.0]	**16.1** **[1.73–355]**	–
Hepatitis B	OR 95 % CI	0.24 [0.03–0.99]	–	0.62 [0.12–2.86]	2.23 [0.74–8.19]	–	0.00	0.00	0.84 [0.04–6.17]	2.75 [0.67–8.80]	–
Hepatits C	OR 95 % CI	12.6 [1.04–368]	–	⁎	⁎	–	0.00	57.7⁎	0.00	0.00	–
Syphilis	OR 95 % CI	0.64 [0.29–1.30]	–	2.48 [0.91–7.97]	**6.89** **[2.83–20.08]**	–	0.00	**6.09** **[1.60–19.3]**	1.40 [0.44–4.05]	2.08 [0.89–4.54]	–
Underweight	OR 95 % CI	**1.89** **[1.24–2.85]**	–	1.08 [0.68–1.72]	1.09 [0.70–1.73]	–	0.96 [0.15–3.64]	2.18 [0.75–5.61]	1.45 [0.75–2.77]	**1.76** **[1.05–2.90]**	

⁎ sample size too small for parametric analysis.

The analysis found some independent associations among the risk factors included in the analysis (sex, age, time from HIV diagnosis, tobacco consumption, alcohol consumption and overweight/obesity) and the diseases and conditions studied. Having one or more comorbidity was associated with male sex (OR = 1.48, 95 % CI: 1.05, 2.09), age > 50 years (OR = 2.69, 95 % CI: 1.92, 3.78), and with alcohol consumption (OR = 1.69, 95 % CI: 1.08, 2.67).

Males were more likely to be underweight (OR = 1.89, 95 % CI: 1.24, 2.85). Both age and the time since HIV diagnosis were found to correlate with the risk of syphilis: patients >50 years showed a higher risk of developing syphilis (OR = 6.89, 95 % CI: 2.83, 20.08) and patients with a shorter duration since HIV diagnosis had higher syphilis prevalence (OR = 6.09, 95 % CI: 1.60, 19.3). An association between a shorter time since HIV diagnosis and Hepatitis C Virus (HCV) was observed; however, the extremely small sample size (*n* = 3) makes this analysis unreliable for parametric methods like logistic regression. Similarly, the link between alcohol consumption and suspected TB (OR = 17.0, 95 % CI: 1.82, 375) is uncertain due to a broad confidence interval. Conversely, as shown in Table 1, women had significantly higher risk of reduced eGFR compared to men, with a greater prevalence of moderate and severe reductions in eGFR (38 % and 1.1 % in females, respectively, compared to 16 % and 0.4 % in males).

For NCDs, age over 50 years emerged as an independent risk factor for all the conditions studied (hypertension, diabetes, and dyslipidemia). Overweight status was associated to hypertension (OR = 2.49, 95 % CI: 1.76, 3.54) and to diabetes (OR = 3.66, 95 % CI: 1.42, 10.02) but not to dyslipidemia. Alcohol consumption was associated with underweight (OR = 1.76, 95 % CI: 1.05, 2.90). No significant associations were found between tobacco or alcohol consumption and any other comorbidity.

**Fig. 3:**
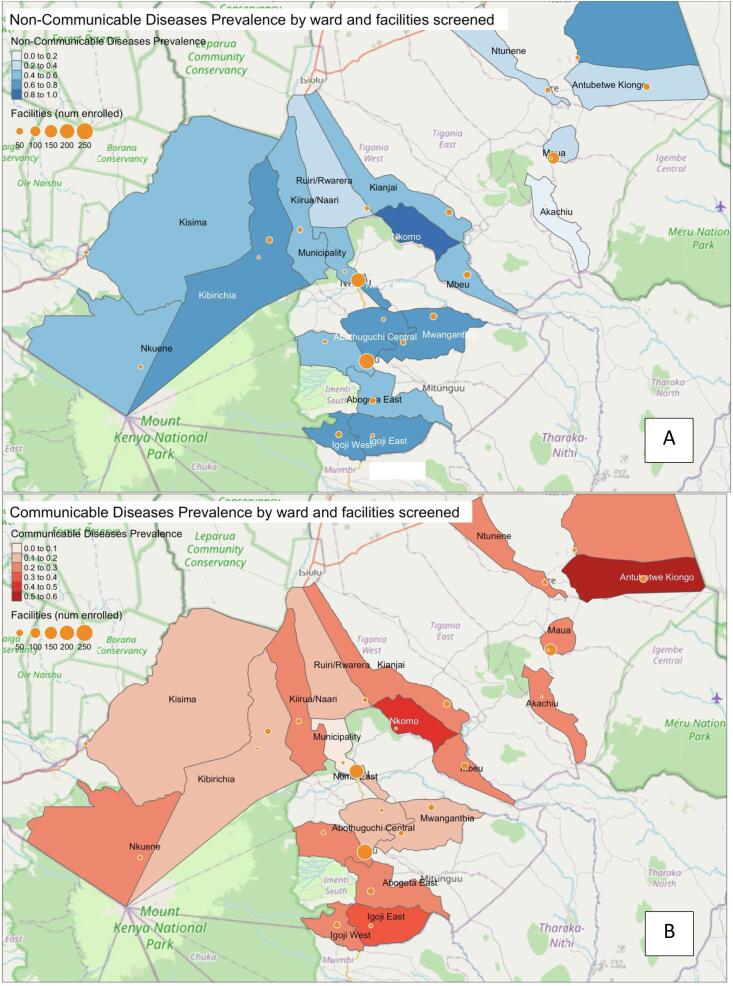
Prevalence of non-communicable diseases (A) and communicable, matenal, neonatal and nutritional conditions (B) in different districts in Meru County.

The geographic distribution of disease prevalence among the HIV-positive cohort is depicted in the Fig. 3, highlighting the disparity across Meru County. The map B shows a higher concentration of communicable diseases in the central and northeastern regions, with a notable prevalence exceeding 0.15 in certain wards. In contrast, the map A representing NCDs prevalence exhibits a more dispersed pattern, with significant prevalence rates (>0.20) spread across multiple wards, including both central and peripheral areas.

## Discussion

Our study investigates the prevalence of comorbidities among HIV-positive patients in Meru County, Kenya, shedding light on the dual burden of disease experienced by this population. The results indicate a significant presence of comorbidities in patients undergoing HIV treatment, with a notable majority of long-term treated patients having at least one other pathology in addition to HIV infection. Specifically, 55.4 % of the HIV-positive individuals in our cohort were found to have comorbidities.

It is important to note that approximately 75.3 % of our sample consisted of women, which does not perfectly reflect the national distribution of HIV-positive patients. According to recent UNAIDS data, women account for about 65.6 % of the adult HIV population in Kenya [12]. However, our sample represents the situation in healthcare centers, where women tend to have a greater presence, highlighting the actual gender-related burden on the county's healthcare system, as known from previous researches [26].

Among our patients, NCDs were particularly prevalent, afflicting 38.5 % of the patients, while communicable diseases were present in 14.9 %.

Notably, dyslipidemia (21.22 %) and hypertension (20.17 %) were the most common NCDs observed. A recent paper found a similar prevalence of hypertension in Kenya, but with lower rates in HIV-positive patients compared to HIV-negative ones (16 % vs. 27 %) [ 27]. A metanalysis examining prevalence of hypertension in East Africa also found a pooled prevalence of hypertension among patients living with HIV in the region of 19.75 % [ 28]. Our results are slightly higher (20.17 %); however, both sets of data suggest a high prevalence of hypertension in this population.

Additionally, communicable diseases such as syphilis (5.23 %), hepatitis B (2.19 %), and hepatitis C (0.29 %) were also prevalent. These findings underscore the complex healthcare landscape in Meru County, highlighting the multifaceted challenge of managing comorbidities among HIV-positive individuals, necessitating tailored strategies and interventions.

This study underscores the high prevalence of comorbidities among HIV-positive patients in care in Kenya, which is consistent with similar observations in other countries within the region. Variability in prevalence, as reported in different studies, is linked to study designs, populations, and healthcare settings. Data from Bloomfield and colleagues show much lower rates of NCDs in Kenya HIV patients; however, it is important to note that these data pertain to a population from 2009, which could explain the observed discrepancies [ 29]. More recent samples from Rajagopaul and Naidoo observed a 23.6 % comorbidity rate in South Africa [ 30], while Uwanyirigira et al. and Eckman et al. highlighted high hypertension and diabetes prevalence in Rwanda and west-Africa in general [ 31,32]. The higher comorbidity risks in older HIV patients, as noted by Roomaney, Van Wyk, & Pillay-van Wyk and Ciccacci, and confirmed in the present analysis, underscore the necessity for age-specific healthcare strategies [ 11,33].

In Addis Ababa, Ethiopia, Demissie et al. reported a 5.1 % prevalence of syphilis among HIV patients, with hepatitis C and B prevalences at 2.9 % and 5.1 %, respectively [ 34]. Comparatively, in Botswana, Zhang et al. found the prevalence of hepatitis C and B among HIV patients to be around 1.54 % and 0.43 % [ 35]. These findings, in comparison with our results, serve as benchmarks for assessing the burden of viral infections in HIV patients across Africa [ 31].

Our study also highlights important gender differences in kidney disease among HIV-positive patients. Women in our cohort had a higher prevalence of eGFR reduction compared to men, which aligns with broader evidence suggesting that women are at increased risk of kidney disease [ 36,37]. Specific challenges, such as pregnancy, can exacerbate disparities. Additionally, HIV itself is a known risk factor for kidney disease, including HIV-associated nephropathy, which is particularly common in individuals of African descent [ 38]. Despite these observations, there is a lack of studies analyzing sex differences in renal disease among HIV patients, underscoring the need for more research to better understand and address the unique health needs of women with kidney disease, particularly those living with HIV in African contexts.

The increasing prevalence of NCDs in Africa, as highlighted by many authors over the past few decades, signifies a critical epidemiological transition [ 7,39,40]. This transition, characterized by the simultaneous burden of rising NCDs alongside persistent communicable diseases, is detailed by Gouda et al. [6], who emphasize the growing impact of NCDs in sub-Saharan Africa. These trends underscore the need for tailored public health strategies and policies, particularly in rapidly urbanizing areas where lifestyle changes, such as shifts in diet and physical activity, contribute to the increasing burden of NCDs. For individuals with HIV in Africa, the extension of life expectancy thanks to improved HIV treatment has introduced additional health challenges, including a heightened risk of NCDs. This evolving health landscape necessitates ongoing research and adaptations within healthcare systems to address the dual burden of HIV and NCDs effectively.

Our study's multivariate analysis reveals age-specific health outcomes and the heightened risk of conditions like hypertension and diabetes, echoing findings by many authors [ [41], [42], [43]]. Notably, our findings did not show a significant link between tobacco/alcohol use and NCDs, possibly due to underreporting, indicating a direction for future research and targeted interventions.

The description of disease prevalence across Meru County revealed variations in the presence of communicable diseases in different regions. These variations suggest that certain areas may face unique challenges. Meanwhile, the widespread prevalence of NCDs across various regions indicates that these health issues are present throughout the county, potentially transcending local geographical and socio-economic differences. This finding highlights the need for an integrated healthcare approach, capable of addressing the diverse health challenges evident in our study population. Further investigation into possible spatial patterns could provide valuable insights into the factors contributing to the higher or lower prevalence of specific diseases in particular areas. Such research would be instrumental in tailoring healthcare strategies to the unique needs of each region within Meru County.

Our study encompasses certain limitations that should be considered in future research. Firstly, our analysis did not include measures like waist circumference, which is vital for assessing metabolic syndromes in HIV-positive patients. The absence of an HIV-negative control group also constrains our ability to fully understand the unique comorbidity burden attributable to HIV, presenting an opportunity for future comparative studies. Additionally, the data on lymphocytes T CD4+ counts were often missing in patient records, despite being expected according to national guidelines. This data is essential for evaluating the stages of HIV infection and its management.

An additional limitation of our study is the lack of specific data on TB diagnosis among HIV-positive patients. We only have information on patients screened for TB using the four-symptom screening method according to local guidelines and referred for TB diagnostics. However, actual diagnosed TB cases data, which could have provided deeper insights into TB incidence and management in this population, is not available.

Importantly, our study is not a population-based prevalence study but focuses exclusively on patients attending HIV care centers. This aspect aligns with the study's primary public health objective, which was to determine the prevalence of comorbidities among patients with HIV attending health centres. However, it is crucial to note that these findings may not be generalizable to the broader HIV-positive population not engaged in regular healthcare. This limitation underscores the need for more extensive research to provide a comprehensive view of the comorbidity burden in the wider HIV-positive population.

Additionally, we didn't fully investigate socioeconomic and behavioral factors, which are important for health outcomes. Alcohol and tobacco use were self-reported, which introduces potential bias, especially for women who may feel societal pressure not to drink or smoke, potentially leading to underreporting. This should be noted as a limitation of our study. Future studies should include these aspects and use a longitudinal approach to track comorbidity progression. Incorporating qualitative research, like patient interviews, could also provide valuable insights into the experiences and challenges of HIV-positive individuals, enhancing our understanding of their health impacts.

The results from the CHAO study in Meru County, Kenya, highlight a significant comorbidity burden among HIV-positive patients, demonstrating the prevalence of both non-communicable and communicable diseases. This finding underscores the urgent need for healthcare strategies tailored to the complex needs of this population, considering age, gender, lifestyle, and HIV status, to enhance HIV care and public health in Kenya.

Notably, our study population largely consisted of patients who had been stable on long-term antiretroviral therapy, a common scenario in many African countries affecting hundreds of thousands of individuals. In our study, these patients were observed to have a comorbidity in more than half of the cases, and a high prevalence of non-communicable diseases. This emphasizes the critical importance of integrating and actively promoting systematic screening and treatment for these conditions, particularly hypertension and dyslipidemia, within HIV programs. Our findings advocate for a comprehensive approach in HIV care that addresses the growing burden of non-communicable diseases in this population, ensuring holistic health management.

## Funding

This research was funded by the Italian Cooperation Agency (AICS) through the “Global Fund” initiative (AID 012168/02/1). The grant was awarded to the 10.13039/501100007642University of Rome Tor Vergata, in partnership with the Community of Sant'Egidio.

## CRediT authorship contribution statement

**Fausto Ciccacci:** Writing – review & editing, Writing – original draft, Methodology, Funding acquisition, Conceptualization. **Benjamin Welu:** Writing – original draft, Investigation, Data curation. **Harrison Ndoi:** Investigation, Data curation. **Claudia Mosconi:** Writing – review & editing. **Carolina De Santo:** Writing – review & editing. **Anna Maria Doro Altan:** Conceptualization, Writing – review & editing. **Joseph Murungi:** Writing – review & editing, Validation. **Koome Muthuri:** Writing – review & editing, Validation. **Mariagrazia Cicala:** Formal analysis, Data curation. **Giovanni Guidotti:** Supervision, Project administration. **Stefano Orlando:** Writing – review & editing, Supervision, Funding acquisition, Formal analysis, Conceptualization.

## Declaration of competing interest

We declare no competing interests.

## Data Availability

Data from this study, including deidentified individual participant data and a comprehensive data dictionary defining each field in the dataset, will be available upon reasonable request. The deidentified participant data will be shared in the form of an Excel spreadsheet with a randomly assigned identifier for each participant to ensure privacy. Additionally, the informed consent forms, which are physically stored on-site, may be digitized and shared upon request, always adhering to patient privacy protections. The study protocol and ethical committee approvals are available at this link: https://www.chaoproject.com/the-project/. These documents will be made available starting from the date of publication and will remain available indefinitely. Access to the data will be granted following a request to the corresponding author who will review requests for scientific and non-commercial research purposes. Requestors will be required to sign a data access agreement to ensure the data's use is in line with ethical standards and privacy protections. If applicable, software code used for analysis may also be shared under similar conditions to support transparency and reproducibility of the findings.
